# Diabetic mastopathy: about two cases

**DOI:** 10.11604/pamj.2024.48.28.42960

**Published:** 2024-05-29

**Authors:** Karam Harou, Amirath Adoufè Sanni, Illyass Essaoudy, Soukaina El Aziz, Abderrahim Aboulfalah, Hamid Asmouki, Abderraouf Soummani

**Affiliations:** 1 Obstetrics and Gynecology Department, Mohammed VI University Hospital Center, Faculty of Medicine and Pharmacy, Marrakech, Morocco

**Keywords:** Mastopathy, diabetes, insulin, case report

## Abstract

Diabetic mastopathy is a rare and benign pathology affecting young individuals with type 1 diabetes or autoimmune diseases. It clinically resembles breast cancer, necessitating a histological examination for a definitive diagnosis. These cases underscore the diagnostic challenges and the importance of histological examination. This report details two cases of diabetic mastopathy at Mohammed VI Hospital in Marrakech. The first case involved a 35-year-old with type 1 diabetes and mastodynia, revealing a 4 x 3 cm nodule in the left breast. Biopsies confirmed fibrous breast tissue with lymphocytic infiltrates, characteristic of diabetic mastopathy, with no recurrence during follow-up. The second case featured a 38-year-old with trisomy 21 and type 1 diabetes presenting with a right breast abscess. Drainage revealed lymphocytic infiltrates, confirming diabetic mastopathy. Though diagnostically challenging, diabetic mastopathy lacks a direct link to breast cancer. Long-term cancer risks in affected patients mirror the general population.

## Introduction

Diabetic mastopathy is an uncommon anatomical and clinical condition, constituting less than 1% of benign breast lesions [1]. A rare benign fibrotic disease of the breast can develop in patients with longstanding and often uncontrolled diabetes mellitus, characterized by the clinical presentation of an irregular, firm, palpable mass that may occur singularly or multiply in one or both breasts. The etiology remains unclear; the disease's autoimmune basis is strongly suspected. The disease primarily affects young and middle-aged women who have had type I diabetes for an extended period; its incidence varies from 0.6% to 13% [2]. Notwithstanding a considerable increase in our knowledge of diabetic mastopathy gained in recent years and several new hypotheses, the pathogenesis of this disease remains unclear [3]. It occurs most often in individuals with diabetes taking long-term insulin or in individuals with diabetes with degenerative complications. This study aimed to report two cases of diabetic mastopathy while explaining the diagnostic challenges and the treatment of this pathology.

## Patient and observation

### Patient 1

**Patient information:** patient aged 35, nulligest, with no family history of breast cancer, followed for 15 years of type 1 diabetes on insulin with total control of her diabetes. She presented for consultation at the hospital for mastodynia.

**Figure 1:**
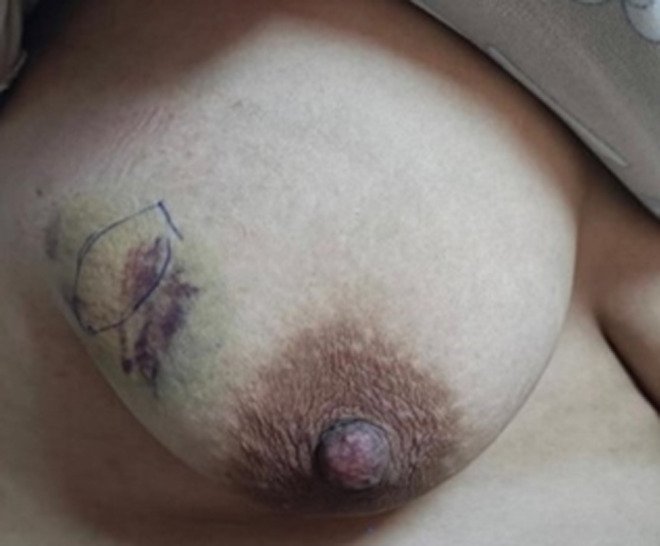
left upper outer quadrant (QSE) nodule of the right breast

**Clinical findings:** physical examination showed a nodule of 4 x 3 centimeters, located in the left upper outer quadrant (QSE) and mobile concerning the deep and superficial planes (Figure 1). There was no palpable axillary lymph node. Examination of the contralateral breast was normal.

**Timeline of the current episode:** she presented with left mastodynia, which had been present for 2 months. March, 7^th^ 2023: gynecology consultation. March, 13^th^ 2023: mammography albumin-to-creatinine ratio (ACR 4) and breast ultrasound (ACR 4). March, 24^th^ 2023: biopsy and histology studies were conducted and concluded to dystrophic breast parenchyma. April 11^th^ 2023: wide excision of the tumor, biopsy, and histology studies concluded diabetic mastopathy.

**Figure 2:**
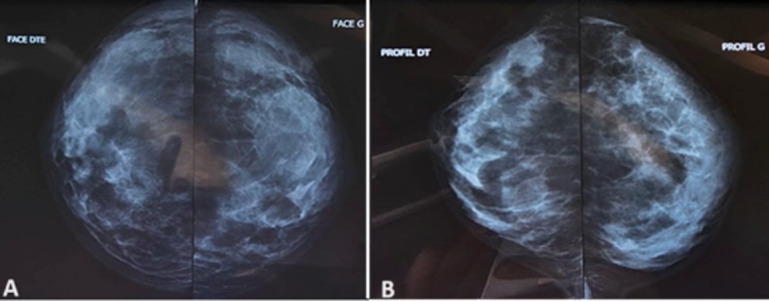
profile and front mammography (A, B)

**Diagnostic assessment:** mammography coupled with breast ultrasound showed dense breasts with no regular area of the left QSE of two sizes: 25 x 18 mm and 20 mm x 20 mm classified ACR 4 without microcalcification or architectural distortion (Figure 2). Histological examination showed after a micro biopsy a dystrophic breast parenchyma with no sign of malignancy. We subsequently carried out a wide excision of the tumor. The final pathological examination revealed fibrotic breast parenchymatous with a lymphocytic infiltrate peri-lobular, peri-ductal, and perivascular with no sign of malignancy suggesting diabetic mastopathy.

**Diagnosis:** the results especially the histological examination was consistent with diabetic mastopathy.

**Therapeutic interventions:** the patient underwent surgical treatment, which involved wide excision of the tumor.

**Figure 3:**
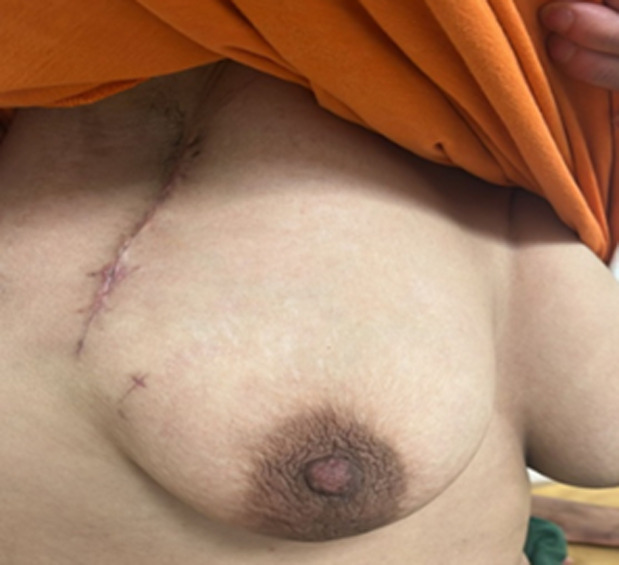
breast 1 month after surgery

**Follow-up and outcome of interventions:** immediate postoperative follow-up took place without complications and the long-term result showed no recurrence (Figure 3).

**Informed consent:** the patient gave informed consent for the entire process from diagnosis to surgical procedure.

### Patient 2

**Patient information:** a 38-year-old female patient, nulligest with Down syndrome, no family history of breast cancer, type 1 diabetic on insulin for 12 years with no control of her diabetes. The patient came to the hospital for a breast abscess.

**Clinical findings:** physical examination revealed a warm, painful, fluctuating collection measuring 3 x 2 centimeters at the lower quadrants and abscessed with skin necrosis; presence of a 2-centimeter right axillary adenopathy. Examination of the contralateral breast was normal.

**Timeline of the current episode:** she consulted for a right breast abscess that had been evolving for 20 days. November, 12^th^ 2023: consultation in the emergency for an inflamed breast. Performing a breast ultrasound performing a breast ultrasound followed by surgery for drainage of the abscess with sampling. Biopsy, histology, and immunohistochemical studies were conducted. November, 25^th^ 2023: the biopsy result concludes with diabetic mastopathy.

**Diagnostic assessment:** ultrasound examination revealed a 32.5 x 25 mm collection straddling the right lower mammary quadrants, with multiple right axillary lymph nodes, the largest of which was 19 x 13 mm. A specimen was taken for histological examination and showed a dense lymphocytic infiltrate of B phenotype (diabetic mastopathy).

**Diagnosis:** the results especially the histological examination was consistent with diabetic mastopathy.

**Figure 4:**
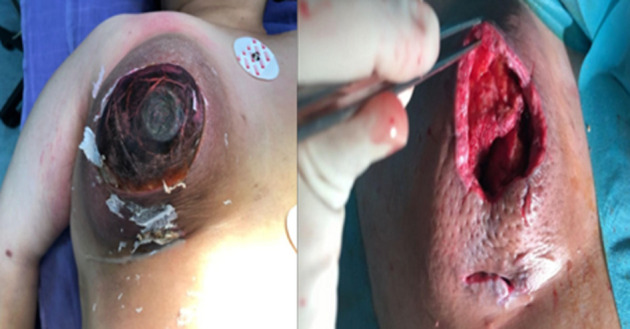
inflammatory breast and necrosectomy

**Therapeutic interventions:** the patient underwent surgical treatment. The abscess was drained, and a necrosectomy was subsequently performed (Figure 4).

**Follow-up and outcome of interventions:** immediate postoperative follow-up occurred without any complications, and the long-term outcome revealed no recurrence.

**Informed consent:** the patient gave informed consent for the entire process from diagnosis to surgical procedure.

## Discussion

Diabetic mastopathy, alternatively referred to as fibrous breast condition or sclerosing lymphocytic lobulitis, is a newly recognized, uncommon, and non-cancerous medical condition that can impact individuals of all genders regardless of age. It predominantly manifests in females and rarely in males, particularly those who have diabetes and are undergoing insulin treatment [4]. The typical age range of affected individuals is between 20 and 40 years old, with type 1 diabetes being the prevailing form [5], as observed in the cases presented in our study.

It´s pathogenesis remains uncertain, Tomaszewski *et al*. suggested that the fibro-inflammatory alterations identified in the breast may be linked to hyperglycemia, which triggers an expansion of the extracellular matrix, followed by an increase in collagen synthesis and a decrease in its breakdown [1]. The proteins undergoing non-enzymatic glycosylation serve as antigens, prompting the autoimmune proliferation of B-lymphocytes and the generation of autoantibodies. The secretion of cytokines leads to the enlargement of epithelial cells within the matrix and the development of specific epithelial cells enmeshed in dense fibrous tissue, known as epithelial fibroblasts (EFBs). The autoimmune aspect of the disease is strongly implicated, as analogous anatomical lesions have been reported in other autoimmune pathologies like Hashimoto's thyroiditis, Sjogren's syndrome, lupus, or insulin-dependent type 2 diabetes [4]. The suggestion regarding insulin-dependent type 2 diabetes may arise from the body's inflammatory response to exogenous insulin [6].

In a clinical context, diabetic mastopathy can be confused with breast cancer, presenting as either a single or multiple, uni- or bilateral, newly formed breast lump with irregular contours and a solid or stony consistency. Typically painless, it grows fast, moves concerning both superficial and deep planes, and can appear anywhere in the breast. Most frequently, it occurs behind the nipple, though its size varies. One of our patients had a single nodule measuring 4cm, poorly defined, with a stony consistency and a neoplastic appearance, and the second one presented with an inflammatory breast. Clinically, these lesions can be confused with breast cancer granulomatous mastitis, or even breast tuberculosis, and it is difficult to distinguish these entities by simple physical examination. For this reason, further investigations are essential.

Medical imaging gives limited information; mammograms often reveal dense breasts, and irregular opacities but never structural distortion or clusters of micro-calcifications [7]. One of our patients had dense BIRADS IV breasts, while the second patient could only benefit from breast ultrasound without mammography, because of the emergency and the pain associated with the breast abscess.

Biopsy remains the preferred paraclinical exam to confirm the diagnosis [4]. Surgical biopsy is not recommended due to increased fibrosis; thus, core biopsy stands as the diagnostic gold standard. The sampled lesion offers ample material for definitive histological assessment [8]. Histological examination reveals three elementary lesion types, often associated: (a) lymphocytic lobulitis characterized by a more or less dense mononuclear inflammatory infiltrate comprising small lymphocytes, notably B lymphocytes, plasma cells, and histiocytes. Predominantly localized in mammary lobules, it may also extend to the perivascular and occasionally perianal areas; (b) consistent, homogenous, dense fibrosis of the stroma; (c) sporadically distributed stromal epithelioid cells.

Our study identified these same histological lesions. The treatment options are limited to diabetes management and surgery. Surgical treatment involves a wide excision of the nodule due to suspicious lesions for malignancy on mammography and micro-biopsies with histopathological examinations not revealing any signs of malignancy. The surgical management take into consideration the ratio between breast volume, tumor size and respect of aesthetics rules for best cosmetic results as it considerate as benign disease for best quality of life. Ricart Selma V *et al*. in a case series involving 6 patients, chose surgical treatment, similar to our study [9]. Spontaneous regression has been reported in the literature. The long-term prognosis selected for breast cancer risk is still unknown.

## Conclusion

Diabetic mastopathy is a rare, benign condition that occurs mainly in young patients with type I diabetes. Clinically, it mimics breast cancer. It´s important to master the diagnostic process and the therapeutic management. Our patients in this case report had an excellent clinical outcome and they did not develop breast cancer.
